# Temporal and Spatial Expression Analysis of Shoot-Regeneration Regulatory Genes during the Adventitious Shoot Formation in Hypocotyl and Cotyledon Explants of Tomato (CV. Micro-Tom)

**DOI:** 10.3390/ijms21155309

**Published:** 2020-07-26

**Authors:** Myoung Hui Lee, Jiyoung Lee, Eun Yee Jie, Seung Hee Choi, Lingmin Jiang, Woo Seok Ahn, Cha Young Kim, Suk Weon Kim

**Affiliations:** 1 Biological Resource Center, Korea Research Institute of Bioscience and Biotechnology (KRIBB), Jeongeup 56212, Korea; mhlee17@kribb.re.kr (M.H.L.); jiyoung1@kribb.re.kr (J.L.); jeannie@kribb.re.kr (E.Y.J.); csh@kribb.re.kr (S.H.C.); jiang6@kribb.re.kr (L.J.); dntjr0412@kribb.re.kr (W.S.A.); kimcy@kribb.re.kr (C.Y.K.); 2 Department of Bioactive Materials, Chonbuk National University, Jeonju 54896, Korea; 3 Department of Bioenergy Science and Technology, Chonnam National University, Gwangju 61186, Korea

**Keywords:** tissue culture, plant regeneration, shoot formation, genes expression, reproducibility, tomato

## Abstract

Enhancing the competence for plant regeneration in tissue culture studies is an important issue not only for efficient genetic transformation of commercial crops but also for the reproducibility of scientific reports. In this study, we investigated optimization of several tissue culture conditions including plant growth regulators, types and ages of explants, culture densities, and plant position in order to improve the competence of adventitious shoot formation of the tomato (*Solanum lycopersicum* cv. Micro-Tom). In addition, we examined the differential expression of D-type cyclin (*CYCD3-1)* and several shoot regeneration regulatory genes from hypocotyl and cotyledon explants of tomato during shoot organogenesis. A treatment of 1 mg L^−1^ Zeatin and 0.1 mg L^−1^ Indole-3-acetic acid (IAA) in Murashige and Skoog (MS) medium containing 3% sucrose was optimal for adventitious shoot formation from hypocotyl and cotyledon explants. The younger explants exhibited more shoot formation regardless of explant types. Additionally, those closest to the shoot apical meristem produced more shoots compared to the other regions in the hypocotyl and the cotyledon explants. Gene expression of *CYCD3-1,* SHOOT MERISTEMLESS (*STM*), and cytokinin dependent WUSCHEL (*WUS*) was significantly higher in younger explants than in older ones. Furthermore, an increase in *CYCD3-1*, *STM*, and *WUS* expression was evident at the distal part of hypocotyls and the proximal part of cotyledons compared to other regions. These differential gene expression profiles exhibited good agreement with the results of shoot formation obtained from diverse explants of tomato. These results suggest that temporal and spatial gene expression of shoot regeneration regulatory genes plays an important role in enhancing the competence and the reproducibility of adventitious shoot formation from tomato explants.

## 1. Introduction

The tomato (*Solanum lycopersicum* cv. Micro-Tom) is both an economically important vegetable crop on a global scale and a model plant for studying fruit development and fruit ripening. Mutagenesis and genetic mutation methods using *Agrobacterium* transformation by direct organogenesis have been used as key tools to facilitate tomato research. In recent years, the CRISPR/Cas9 genome editing system has been used to develop useful tomato cultivars through in vitro tissue culture [1,2,3,4,5,6,7].

In general, tomato plant regeneration is achieved through adventitious shoot formation from callus derived from various explants [8]. Plant growth regulators may be the most important factors controlling the shoot regeneration capacity of explants [9,10]. Along with plant growth regulators, several tissue culture parameters also play a crucial role in controlling the adventitious shoot regeneration potential of explants, including plant age [11,12,13], explant position [13], explant orientation (abaxial or adaxial) [14,15], and explant density [16,17]. Thus, these complexities of optimal explant preparation significantly decrease the reproducibility in plant tissue culture. To further improve adventitious shoot organogenesis in tomato genotypes, it is essential to control the physiological state of the donor plant. However, as of yet, no comparative studies have been published on the relationships between the types of tomato explants and the expression profiles of major regulatory genes involved in shoot organogenesis.

Plant growth regulators including auxin and cytokinins are key regulators for determining cellular differentiation and dedifferentiation in plant tissue culture studies. Despite the importance of auxin and cytokinins, the underlying molecular mechanisms of their function have only been recently revealed [18,19]. In vitro cell proliferation and plant regeneration require the generation of a new cell mass and cell fate transition. Wounding is required to initiate cell-fate change during shoot regeneration [20]. Wounding induces a rapid influx of calcium ions into cells and a subsequent increase in reactive oxygen species (ROS), activating downstream signaling cascades [21]. WOUND INDUCED DEDIFFERENTIATION 1 (*WIND1*), which encodes the AP2/ERF transcription factor, is a key regulator of wound-induced cellular reprogramming in plants. *WIND1* promotes callus formation and shoot regeneration by up-regulating the expression of the ENHANCER OF SHOOT REGENERATION1 (*ESR1*) gene [22]. ESR1 encodes a member of the ETHYLENE RESPONSIVE FACTOR (ERF) family of transcription factors, and ESR1 overexpression greatly enhances the efficiency of shoot regeneration in *Arabidopsis* tissue culture [23,24]. Cytokinin synthesis and *WIND*-dependent pathways converge on the activation of cytokinin signaling mediated by type-B ARABIDOPSIS RESPONSE REGULATOR 1 (*ARR1*) and *ARR12* [25,26], which leads to cell-cycle re-entry via the up-regulation of D-type cyclin *CYCD3;1* [25]. Cytokinin and the class III homeodomain-leucine zipper (HD Zip III) transcription factor PHABULOSA (*PHB*), PHAVOLUTA (*PHV*), and REVOLUTA (*REV*) are additional regulators required for cytokinin dependent WUSCHEL (*WUS*) induction and subsequent shoot regeneration [27]. HD Zip III up-regulates the homeobox transcription factor SHOOT MERISTEMLESS (*STM*) for shoot meristem formation [28].

In this study, we optimized several tissue culture parameters for adventitious shoot regeneration, including type, age, location, density, and orientation of tomato explants, in the culture medium to establish a more efficient and reproducible shoot-regeneration system in tomato cv. Micro-Tom culture. We also investigated differential gene-expression patterns of the genes that regulate shoot regeneration of tomato explants depending on diverse cultural parameters. The explants of younger plants and the parts closer to the shoot apical meristem demonstrated higher shoot formation rates and higher expression levels of *CYCD3-1*, *STM*, and *WUS* during cultivation. These results provide an efficient and reproducible method for culturing tomato explants and key information of temporal and spatial differences in the expression of shoot-regeneration regulatory genes during adventitious shoot formation.

## 2. Results

### 2.1. Effect of Sucrose Concentration and Plant Growth Regulators on Shoot Regeneration Efficiency

**Figure 1:**
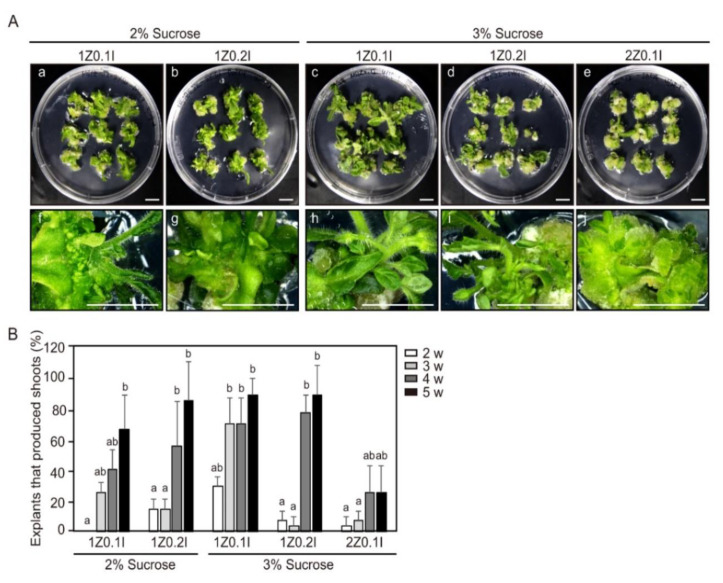
Effect of hormones and sucrose concentration in the shoot induction medium (SIM) for adventitious shoot formation. (**A**) Cotyledon explants of 4-days-after-germination seedlings were cultured in SIM. Plant explants 4 weeks (**a**–**e**) and 5 weeks (**f**–**j**) after incubation. (**a**,**f**) 1 mg L^−1^ Zeatin, 0.1 mg L^−1^ Indole-3-acetic acid (IAA), and 2% sucrose; (**b**,**g**) 1 mg L^−1^ Zeatin, 0.2 mg L^−1^ IAA, and 2% sucrose; (**c**,**h**) 1 mg L^−1^ Zeatin, 0.1 mg L^−1^ IAA, and 3% sucrose; (**d**,**i**) 1 mg L^−1^ Zeatin, 0.2 mg L^−1^ IAA, and 3% sucrose; (**e**,**j**) 2 mg L^−1^ Zeatin, 0.1 mg L^−1^ IAA, and 3% sucrose. Scale bars = 1 cm. (**B**) The efficiency of adventitious shoot formation for different combinations of treatments of Zeatin, IAA, and sucrose. Three independent experiments were performed on 81 explants. Error bars represent SD (N = 81). Different letters on the bars indicate significant differences between each treatment (ANOVA followed by a Tukey’s test, *p* < 0.05). Z = Zeatin; I = IAA; w = weeks.

The culture conditions for adventitious shoot formation from hypocotyl and cotyledon explants of tomato (cv. Micro-Tom) were optimized (Figure 1, Figures S2 and S3). The effects of sucrose concentration (2% and 3%) and combined treatments of Zeatin (1–2 mg L^−1^) and Indole-3-acetic acid (IAA) (0.1–0.2 mg L^−1^) on adventitious shoot formation from cotyledon explants of 4-day-old seedlings were examined. After 2 weeks of incubation, green calli formed on cut edges of cotyledon explants. However, the frequency of green calli formation depended on the combination of Zeatin and IAA treatments. Additionally, new small shoots began to form on the cut edges of cotyledon explants after 2 weeks of incubation. After 3 weeks of incubation, the frequency of adventitious shoot formation was 70% when the cotyledon explants were cultured in MS medium supplemented with 1 mg L^−1^ Zeatin, 0.1 mg L^−1^ IAA, and 3% sucrose. The frequency of adventitious shoot formation from cotyledon explants declined with increasing Zeatin and IAA concentrations. Reducing the sucrose concentration in the culture media did not produce positive effects for adventitious shoot formation from cotyledon explants. In addition, when the sucrose concentration in the culture medium decreased to 2%, vitrified shoot formation increased. After 5 weeks of incubation, the frequency of adventitious shoot formation reached 90%, except for the 2 mg L^−1^ Zeatin, 0.1 mg L^−1^ IAA, and 3% sucrose treatment and the 1 mg L^−1^ Zeatin, 0.1 mg L^−1^ IAA, and 2% sucrose treatments (Figure 1). On the basis of results, we concluded that the optimal culture condition for adventitious shoot formation from tomato explant was in MS medium containing 1 mg L^−1^ Zeatin, 0.1 mg L^−1^ IAA, and 3% sucrose.

### 2.2. Effect of Explant Orientations on Shoot-Regeneration Efficiency

Next, the adaxial orientation and the abaxial orientations were compared to investigate the most efficient orientation. Shoots production in the abaxial orientation was higher than in the adaxial orientation (Figure S2). Nearly 90% and 70% of those in an abaxial orientation produced shoots in 4-day-old and 7-day-old seedlings (DAG4 and DAG7) (hereafter referred to as day-after-germination: DAG4, DAG7, and DAG10), respectively, whereas only 45% of the explants produced shoots in the adaxial orientation in DAG4 and DAG7 after 5 weeks.

### 2.3. Effect of Explant Density on Shoot-Regeneration Efficiency

In addition, to investigate the effect of density on shoot regeneration efficiency, 9–16 cotyledon explants were cultured in the Petri dishes. Statistically significant differences in shoot regeneration among the cultured explants were observed. The best results were seen when nine of the cotyledon explants were cultured per dish, while at 16, the lowest yield occurred, likely as a result of the high competition (Figure S3). Thus, this culture condition was used in a subsequent experiment for optimizing tomato explant and gene expression profiling.

### 2.4. Effect of Explant Ages on Adventitious Shoot Formation of the Tomato

**Figure 2:**
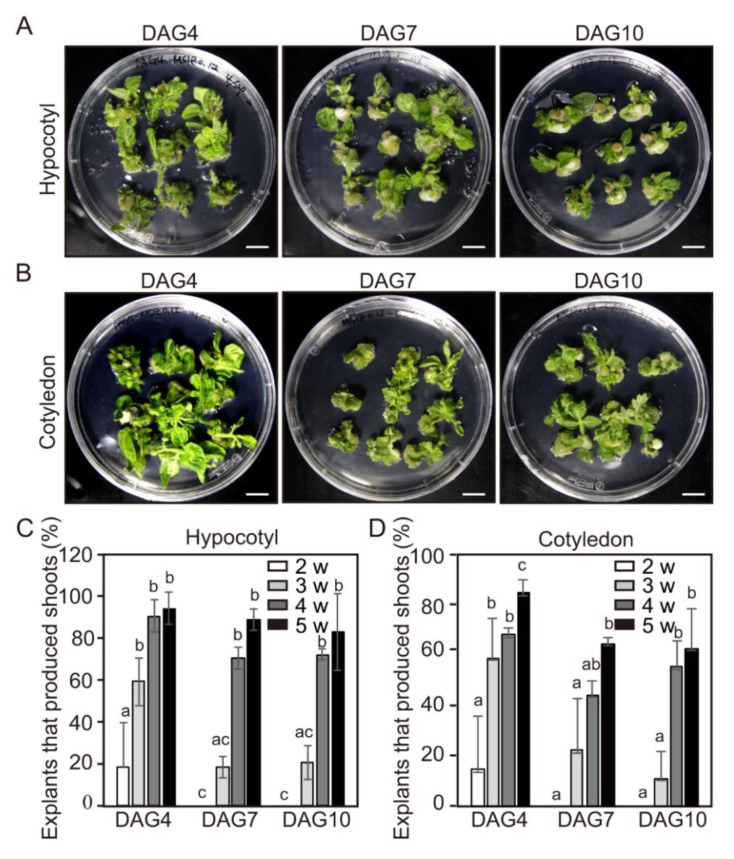
The efficiency of adventitious shoot formation from hypocotyl and cotyledon explants of tomato from seedlings of different ages. (**A**,**B**) Adventitious shoot formation from hypocotyl (**A**) and cotyledon (**B**) explants of DAG4, DAG7, and DAG10 seedlings cultured in the shoot induction medium (SIM) for 4 weeks. Explants of cotyledons for all treatments were placed an abaxial orientation. Scale bars = 1 cm. (**C**,**D**) The efficiency of adventitious shoot formation from hypocotyl (**C**) and cotyledon explants (**D**) of DAG4, DAG7, and DAG10 seedlings examined after 5 weeks of culture in SIM. Data are representative of three independent experiments and values are expressed in mean ± SD (N = 81). Different letters on the bars indicate significant differences between each treatment (ANOVA followed by a Turkey’s test, *p* < 0.05). DAG = day after germination; w = weeks.

To investigate the effect of explant ages on adventitious shoot formation, hypocotyl and cotyledon explants from 4-, 7-, and 10-day-old seedlings (Figure S1A) were incubated in the above-mentioned culture conditions. After 2 weeks of incubation, the frequency of adventitious shoot formation from each explant was examined at one week intervals. The total number of newly formed shoots was counted every week from the second to the fifth week of the culture. The number of morphologically normal shoots was counted, excluding abnormal shapes such as examples with single leaves. After 5 weeks of incubation, over 80% of hypocotyl explants were able to form adventitious shoots regardless of explant ages (Figure 2A,C). As with the hypocotyl explants, over 75% of cotyledon explants could also produce adventitious shoots regardless of explant ages (Figure 2B,D). Therefore, the explant age did not play a crucial role in adventitious shoot formation from hypocotyl and cotyledon explants when the culture period was fully extended. However, if the incubation period was shortened, the frequency of adventitious shoot formation from hypocotyl and cotyledon explants was significantly affected by explant age. Even after 2 weeks of incubation, 20% of hypocotyl explants derived from 4-day-old seedlings were able to form adventitious shoots. However, no adventitious shoot formation was observed from the hypocotyl explants derived from 7- and 10-day-old seedlings under the same incubation period. After 3 weeks of incubation, the frequencies of adventitious shoot formation from hypocotyl explants derived from 4-, 7,- and 10-day-old seedlings were 59%, 18%, and 20%, respectively (Figure 2C). These results clearly show that the younger hypocotyl explants could undergo shoot formation the fastest.

The effect of explant age on adventitious shoot formation from cotyledon explants was similar to that of the hypocotyl explants. Over 15% of cotyledon explants derived from 4-day-old seedlings were able to form adventitious shoots (Figure 2D). However, no adventitious shoot formation was observed from the cotyledon explants derived from 7- and 10-day-old seedlings. After 3 weeks of incubation, the frequencies of adventitious shoot formation from hypocotyl explants derived from 4-, 7-, and 10-day-old seedlings were 59%, 22%, and 11%, respectively (Figure 2D). These results also show that the younger cotyledon explants could undergo shoot formation the fastest

### 2.5. Effect of Positional Difference on Adventitious Shoot Formation Within Individual Tomato Explants

To investigate the effect of positional differences on adventitious shoot formation, three segments of hypocotyl and cotyledon explants derived from 4-day-old seedlings were incubated in the above-mentioned culture conditions. Hypocotyls were divided into three segments from where they were excised, defined as distal, medium, and proximal. The distal segment was from just below the cotyledon and the leaves, while the proximal segment was close to the roots. Cotyledon explants were also divided into three segments from where they were excised as proximal, central, and distal segments (Figure S1B). After 2 weeks of incubation, the frequency of adventitious shoot formation from each hypocotyl and cotyledon segment was examined at weekly intervals. The total number of newly formed shoots was counted every week from the second to the fifth weeks of the culture.

**Figure 3:**
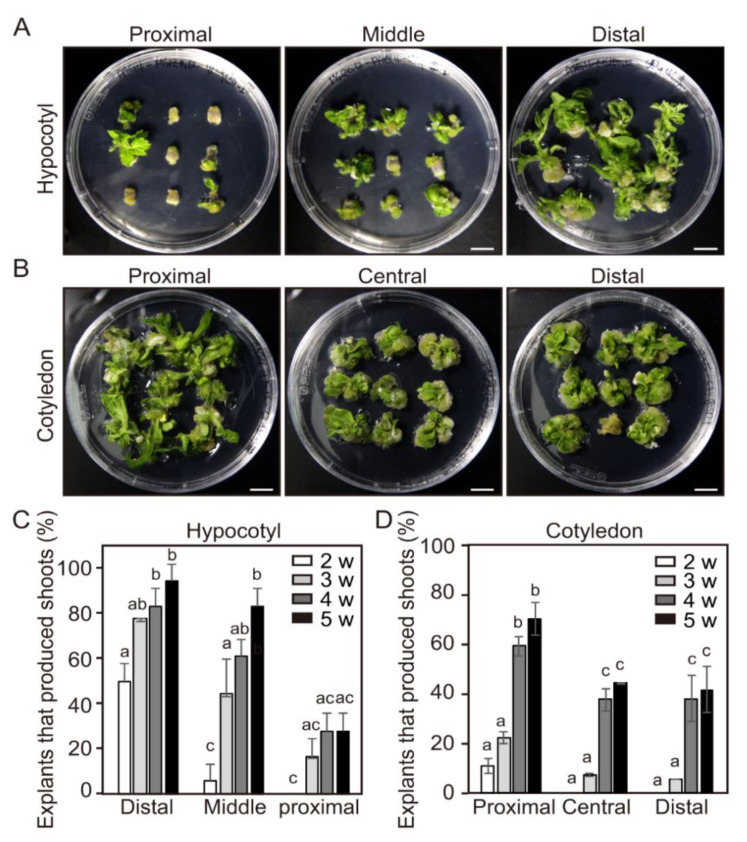
The efficiency of adventitious shoot formation from different segments within individual hypocotyl and cotyledon explants. (**A**,**B**) Adventitious shoot formation at different positions in DAG4 seedlings of hypocotyl (**A**) and cotyledon (**B**) explants incubated for 4 weeks. Scale bars = 1 cm. (**C**,**D**) The efficiency of adventitious shoot formation at different positions of DAG4 hypocotyls (**C**) and cotyledons (**D**) tested in shoot induction medium for 5 weeks. Data are representative of three independent experiments and values are expressed in mean ± SD (N = 81). Different letters on the bars indicate significant differences between each treatment (ANOVA followed by a Turkey’s test, *p* < 0.05). DAG = day after germination; w = weeks.

After 5 weeks of incubation, abundant shoots formed on the distal segments of the hypocotyl; however, the number of shoots was significantly lower in cultures of the middle and the proximal segments (Figure 3A,C). Interestingly, the frequency of adventitious shoot formation increased along the hypocotyl axis of 4-day-old seedlings. As with the hypocotyl explants, the frequency of adventitious shoot formation from the three segments of cotyledon explants also varied significantly depending on position (Figure 3B,D). The frequency of adventitious shoot formation from the proximal segment was much higher than those from the central and the distal segments of the cotyledon explants. The segment closest to the hypocotyl axis produced the most adventitious shoots. These results suggest that different positions within a single explant vary in their potential for adventitious shoot formation.

The initial relative frequencies of adventitious shoot formation from the three segments (distal, middle, and proximal) of hypocotyl explants were 50%, 6%, and 0%, respectively, after 2 weeks of incubation. The relative frequencies of those from the distal and the middle segments reached 94% and 83%, respectively, 5 weeks after incubation (Figure 3C). However, the frequency from the proximal position of hypocotyl explants was lower than the distal position, even though the culture period was extended. These results clearly show that relative position is an important factor for adventitious shoot formation in the explanted hypocotyl.

The initial frequencies of adventitious shoot formation from the three segments (proximal, central, and distal) of cotyledon explants were similar to those of the hypocotyl explants (11%, 0%, and 0%, respectively) after 2 weeks of incubation. After 5 weeks, the relative frequencies had increased to 70%, 44%, and 42%, respectively (Figure 3D). These results show that positional differences also occur in adventitious shoot formation in cotyledon explants but to a less effective extent than that in hypocotyls. We found that the segments close to the shoot apical meristem were more prone to adventitious shoot formation than segments further from them.

### 2.6. Temporal Changes in Expression Levels of CYCD3-1 and Major Shoot-Regeneration Regulatory Genes from Tomato Explants During Adventitious Shoot Formation

Since age and position are important factors involved in shoot formation from hypocotyl and cotyledon explants of tomato, we examined gene-expression profiles of *CYCD3-1* and major shoot-regeneration regulatory genes to verify whether temporal and spatial expression changes are related to the acceleration of adventitious shoot formation.

**Figure 4:**
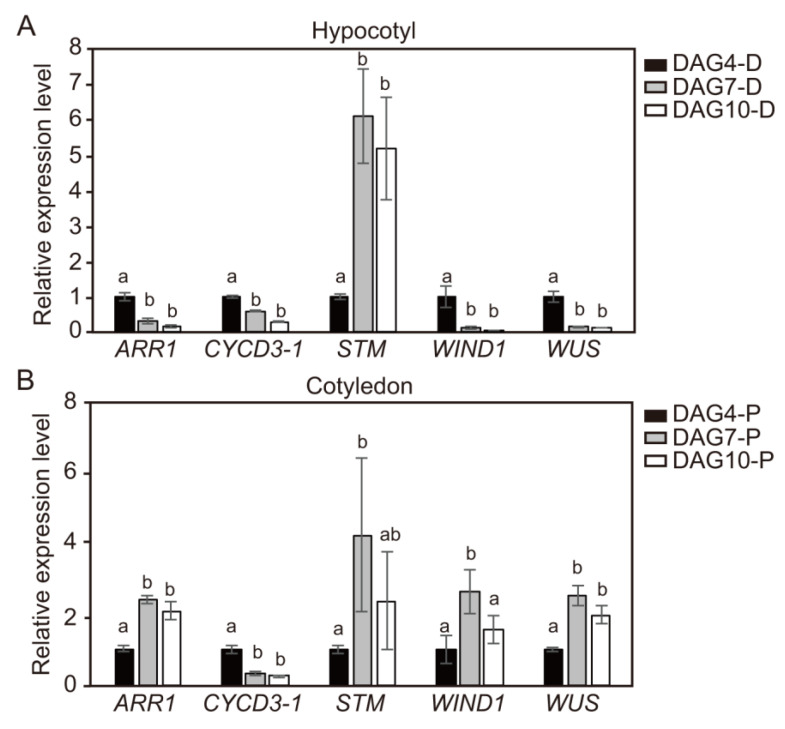
The relative expression levels of *CYCD3-1* and shoot regeneration regulatory genes according to the age of hypocotyl and cotyledon explants seedlings. (**A**) Normalized relative expression of *ARR1*, CYCD3-1, *STM*, *WIND1*, and *WUS* at the distal position of hypocotyls of DAG4, DAG7, and DAG10 seedlings determined using quantitative reverse transcription PCR (qRT-PCR). (**B**) The relative gene expression level of *ARR1, CYCD3-1*, *STM*, *WIND1*, and *WUS* at the proximal position of cotyledon explants of DAG4, DAG7, and DAG10 seedlings. Data are representative of the results from three independent experiments. Error bars represent SD (*n* = 3). Different letters in each gene indicate statistically significant differences the three DAG groups (AVOVA followed by a Turkey’s test, *p* < 0.05). DAG = day after germination; D = distal; P = proximal.

The quantitative reverse transcription PCR (qRT-PCR) analysis showed that the transcripts levels of *ARR1*, *CYCD3-1*, *WIND1*, and *WUS* genes, but not *STM,* from hypocotyl explants of DAG4 were much higher than those of hypocotyl explants from DAG7 and DAG10 seedlings. The transcript levels of *STM* greatly increased in DAG7 and DAG10 hypocotyl explants (Figure 4A). On the contrary, cotyledon explants showed a slightly different expression pattern of these shoot-regeneration regulatory genes depending on explant age. The transcript levels of all the measured shoot-regeneration regulatory genes from the cotyledon explants of DAG4 were slightly lower than those of DAG7 and DAG10 seedlings (Figure 4B). However, the transcript levels of *CYCD3-1* were slightly higher in DAG4 cotyledon explants than DAG7 and DAG10 cotyledon explants. These gene-expression profiles from the DAG4 cotyledon explants seemed contradictory to the shoot formation results (Figure 2).

**Figure 5:**
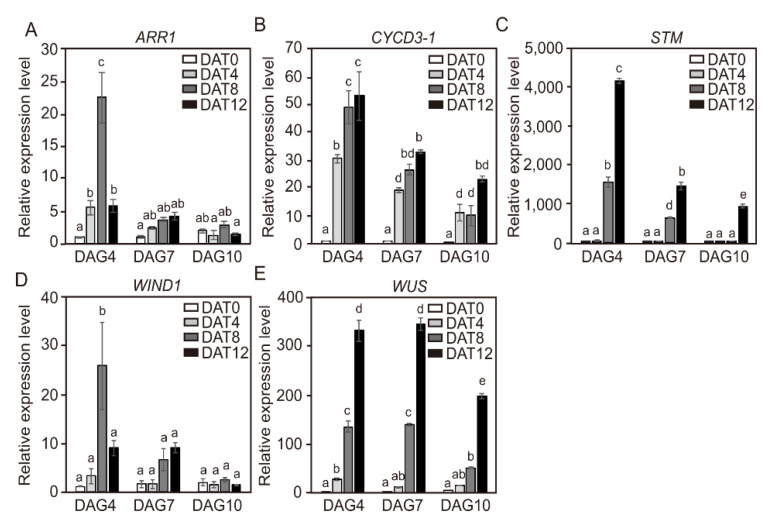
The relative expression levels of *CYCD3-1* and shoot regeneration regulatory genes in the cotyledon explants at different ages for tomato seedlings during incubation in shoot induction medium (SIM). The relative gene-expression level of *ARR1* (**A**), *CYCD3-1* (**B**)*, STM* (**C**), *WIND1* (**D**), and *WUS* (**E**) in the proximal position of cotyledon explants of DAG4, DAG7, and DAG10 seedlings. Data are representative of the results from three independent experiments Error bars represent SD (n = 3). Different letters on the bars indicate significant differences between each treatment (ANOVA followed by a Turkey’s test, *p* < 0.05). DAG = day after germination; DAT = day after treatment.

In order to investigate why DAG4 cotyledon explants demonstrated higher adventitious shoot formation than DAG7 and DAG10 explants despite the low initial transcript levels of shoot regeneration regulatory genes, the levels during incubation in shoot induction medium (SIM) were examined. The transcript levels of *ARR1*, *CYCD3-1*, *STM*, *WIND1,* and *WUS* genes from cotyledon explants of DAG4, DAG7, and DAG10 seedlings were examined at 4-day intervals after the cotyledon explants were transferred to SIM for shoot formation (Figure 5). The transcript levels of three genes (*CYCD3-1*, *STM*, and *WUS)* sharply increased as the incubation time in SIM was extended. Even more unexpectedly, the quantitative increase in transcript levels was much higher in the DAG4 cotyledons than DAG7 and DAG10. Although the initial transcript levels of the *STM* and the *WUS* genes were lower in DAG4 cotyledons than in DAG7 or DAG10, they increased during incubation in SIM (Figure 5B,C,E). However, the transcript levels of *ARR1* and *WIND1* genes from cotyledon explants of DAG4 were highly increased to higher levels at 8 days after treatment, and this was followed by a decrease at DAT12. During incubation in SIM, the transcript level slightly increased in DAG7, but it was not significant compared to DAG4, and the transcript level in DAG10 did not change during culture period (Figure 5A,D).

### 2.7. Spatial Changes in Expression Levels of CYCD3-1 and Major Shoot-Regeneration Regulatory Genes from Tomato Explants During Adventitious Shoot Formation

**Figure 6:**
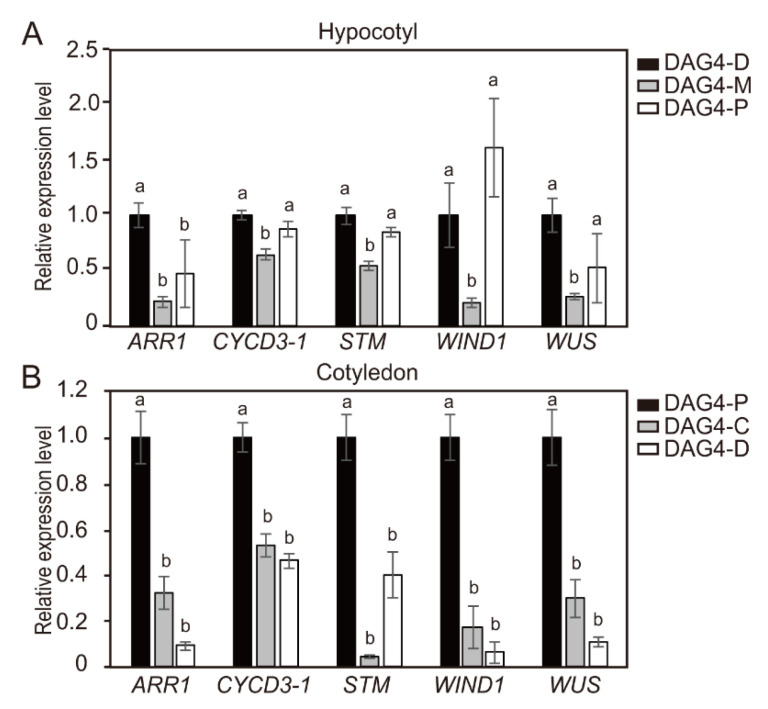
The relative expression levels of *CYCD3-1* and shoot regeneration regulatory genes in hypocotyl and cotyledon explants excised from in vitro-grown seedlings in different positions. (**A**) The relative gene expression levels of *ARR1*, *CYCD3-1*, *STM, WIND1*, and *WUS* in distal (D), middle (M), and proximal (P) positions of DAG4 seedling hypocotyl explants. (**B**) The relative gene expression levels of *ARR1, CYCD3-1*, *STM*, *WIND1*, and *WUS* in proximal (P), middle (M), and distal (D) positions of DAG4 seedling cotyledon explants. Three independent experiments were performed. Error bars represent SD (*n* = 3). Different letters in each gene indicate statistically significant differences the three DAG groups (AVOVA followed by a Turkey’s test, *p* < 0.05). DAG = day after germination.

**Figure 7:**
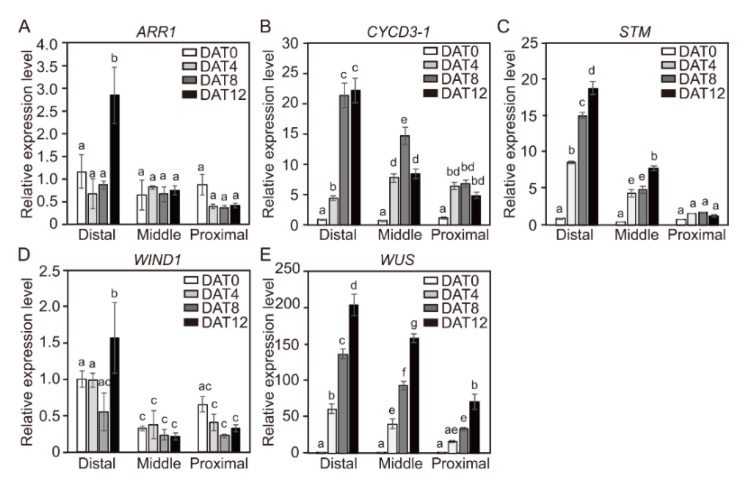
The relative expression levels of *CYCD3-1* and shoot regeneration regulatory genes in hypocotyl explants in different positions for tomato seedlings during incubation in shoot induction medium (SIM). The relative gene expression levels of *ARR1* (**A**), *CYCD3-1* (**B**), *STM* (**C**), *WIND1* (**D**), and *WUS* (**E**) in DAG4 hypocotyl seedling. Data are representative of the results from three independent experiments. Different letters on the bars indicate significant differences between each treatment (ANOVA followed by a Tukey’s test, *p* < 0.05). DAG = day after germination; DAT = day after treatment.

Gene-expression profiling was performed to determine whether changes in the spatial expression of *CYCD3-1* and major shoot-regeneration regulatory genes cause accelerated adventitious shoot-formation in tomato explants. Hypocotyl and cotyledon explants derived from 4-day-old seedlings were dissected into three segments, and the expression levels of shoot-regeneration regulatory genes from each explant were examined. The qRT-PCR analysis revealed transcript levels of *ARR1*, *CYCD3-1*, *STM*, *WIND1*, and *WUS* genes from hypocotyl (Figure 6A) and cotyledon (Figure 6B) explants. Both distal and proximal segments of the hypocotyl explants showed higher expression levels of *CYCD3-1* and shoot-regeneration regulatory genes than the middle segments (Figure 6A). These gene-expression profiling results from the three different segments of DAG4 hypocotyl explants did not match the shoot formation results (Figure 3C). Thus, the transcript levels of the five genes from the three segments were examined at 4-day intervals after each segment was transferred to SIM for shoot formation (Figure 7). Interestingly, the transcript levels of three genes (*CYCD3-1*, *STM*, and *WUS*) slightly declined from the distal to the proximal segments along the hypocotyl axis as the incubation time in SIM was extended. The transcript levels in two other genes (*ARR1* and *WIND1*) were slightly higher in the distal segment, but there was no significant increase in expression during incubation time in SIM between the three segments. These results indicate that *CYCD3-1* and four shoot-regeneration regulatory genes are more highly expressed in the distal segments of hypocotyl than the other segments.

**Figure 8:**
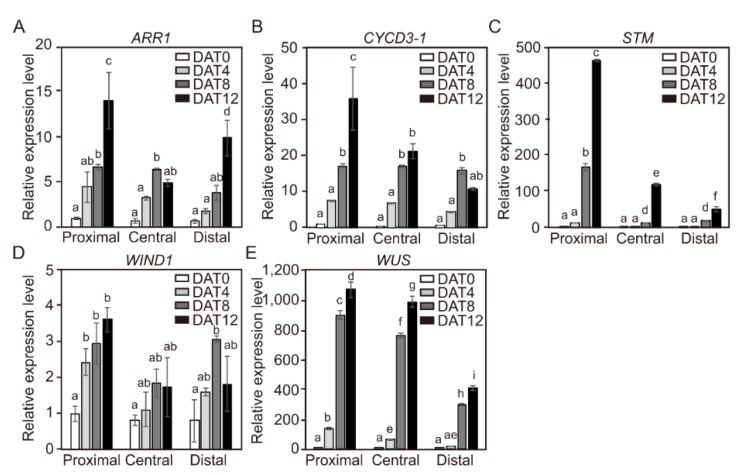
The relative expression levels of *CYCD3-1* and shoot regeneration regulatory genes in cotyledon explants in different positions for DAG4 seedlings during incubation. Relative gene expression level of *ARR1* (**A**), *CYCD3-1* (**B**), *STM* (**C**), *WIND1* (**D**), and *WUS* (**E**) in three positions of DAG4 seedling cotyledon explants. Data are representative of the results from three independent experiments. Error bars represent SD (*n* = 3). Different letters on the bars indicate significant differences between each treatment (ANOVA followed by a Tukey’s test, *p* < 0.05). DAG = Day after germination; DAT = day after treatment.

Positional differences in the expression of the *CYCD3-1* and four shoot-regeneration regulatory genes in the three segments of cotyledons derived from 4-day-old seedlings were also examined (Figure 6B). The transcript levels of all five genes were highest in the proximal segments (Figure 6B). These gene-expression profiling results did not perfectly match the shoot formation results from the three cotyledon segments (Figure 3D). Thus, the transcript levels of the five genes from each of the DAG4 cotyledon segments were also examined at 4-day intervals after being transferred to SIM for shoot formation (Figure 8). The transcripts levels of all five genes gradually increased in the proximal segments as the incubation time in SIM was extended. Similar to the hypocotyl explants, the transcript levels of three genes (*CYCD3-1*, *STM*, and *WUS*) from the cotyledon explants slightly declined from the proximal to the central to the distal segments. The other two genes (*ARR1* and *WIND1*) were highly expressed in the proximal segments of the cotyledon explants, whereas there was no significant difference in expression levels between the central and the distal segments.

## 3. Discussion

Reproducibility is a key limiting factor in the practical application of plant tissue culture research. To increase reproducibility, accurate culture conditions and sample preparation are the most important factors. According to earlier studies, the frequency of adventitious shoot formation from tomato explants is highly dependent on genotypes, types of explants, and supplementation of plant growth regulators [29]. Kaul et al. (2014) reported that the best culture condition for shoot regeneration from cotyledon explants of tomato (cv. Pusa Early Dwarf; PED) was the treatment of 1 mg L^−1^ Zeatin, 0.2 mg L^−1^ IAA, and 2% sucrose [30]. In the case of tomato (cv. S-22), maximum shoot formation was observed using a treatment of 1.2 mg L^−1^ Zeatin, 0.2 mg L^−1^ IAA, and 3% sucrose [31]. In addition, shoot regeneration of cotyledon in two types of tomato (cv. Zuiken and cv. Ailsa Crai) was tested in 1 mg L^−1^ Zeatin, 0.1 mg L^−1^ IAA, and 3% sucrose [32,33]. In this study, the optimal culture conditions for adventitious shoot formation from cotyledon explants of tomato (cv. Micro-Tom) were similar to those published in earlier reports. Combining these results, we determined that the optimal culture conditions for adventitious shoot formation from hypocotyl and cotyledon explants of tomato (cv Micro-Tom) were MS medium containing 1 mg L^−1^ Zeatin, 0.1 mg L^−1^ IAA, and 3% sucrose (Figure 1).

In plant tissue culture, the age of the explants is also a significant factor affecting the efficiency of genetic transformation and plant-regeneration potential [11]. Yildiz (2012) reported that 7-day-old flax (*Linum usitatissimum* L.) cv. “1886 Sel” seedlings showed the highest shoot-regeneration efficiency among different ages of explants (7, 12, and 12 days) [13]. In *Cucumis sativus*, cotyledon explants from younger seedling (6 h~21 h imbibition) gave a significantly higher shoot formation than older seedling (3-day imbibition) [34]. In this study, we also found that the frequencies of adventitious shoot formation from hypocotyl explants derived from 4-, 7-, and 10-day-old seedlings were 59%, 18%, and 20%, respectively, after 3 weeks of incubation (Figure 2C), and that the frequency of adventitious shoot formation from cotyledon explants was similar to that of hypocotyl explants (Figure 2D). These results show that the younger hypocotyl and cotyledon explants undergo shoot formation faster than the older ones. Considering these results, we inferred that the competence period required for shoot formation could be shorter in younger explants than older ones.

The position is also an important factor affecting the regeneration potential of explants, i.e., shoot-regeneration efficiency varies depending on the particular region of an individual explant. Yildiz (2012) reported that shoot-regeneration efficiency is the highest in the upper part of hypocotyls [13]. In *Cucumis sativus*, proximal regions of cotyledon explants produced more shoots than distal parts [34]. Similarly, we found that the frequencies of adventitious shoot formation from the three segments (distal, middle, and proximal) of hypocotyl explants were 50%, 6%, and 0%, respectively after 2 weeks of incubation (Figure 3C). Interestingly, the region closest to the hypocotyl axis produced the most adventitious shoots. The positional differences were also observed in cotyledon explants where the relative frequencies from the three segments (proximal, central, and distal) reached 70%, 44%, and 42%, respectively, if the culture period was extended to 5 weeks (Figure 3D). We found that the distal region of the hypocotyl and the proximal regions of the cotyledon was more prone to adventitious shoot formation than the other regions of the same explants. These results imply that the shoot-regeneration efficiency of tomato explants can vary depending on region. However, we were unable to determine the exact cause of the spatial differences in shoot-regeneration efficiency. The exact role of endogenous growth regulators in adventitious shoot formation is still unclear, but they are known to play a crucial role in the regulation of in vitro shoot development. Auxin is generally synthesized in young leaves and shoot apical meristems and then transported basipetally in the stem. Recently, Hu et al. (2017) reported that polar auxin transport inhibited shoot organogenesis at the basal end of epicotyl segment explants of citrus due to the accumulation of endogenous auxin [35]. In explants treated with the auxin transport inhibitor N-1-naphthylphthalamic acid (NPA), citrus stem explants showed shoot development at both the apical and the basal ends [35]. Thus, temporal and spatial differences in shoot-regeneration efficiency from tomato explants shown here might be due to differences in endogenous auxin levels in each explant. Considering these results, we suggest that adventitious shoot formation can be greatly increased if the optimal temporal and the spatial parameters are selected during explant preparation. Therefore, our results could be directly applied to allow increased adventitious shoot formation as well as reproducibility of shoot formation from tomato explants.

Further useful data came from our study of the transcript levels of shoot-regeneration regulatory genes involved in adventitious shoot formation. We found that the efficiency of shoot formation increased in explants or segments with high gene expression. The distal and the proximal segments of hypocotyl explants showed higher expression levels of *CYCD3-1* and four shoot-regenerative regulatory genes than the middle segments (Figure 6A). However, as the incubation time in SIM medium increased, the transcription of *CYCD3-1*, *STM*, and *WUS* genes resulted in higher expression levels at the distal region of the axis (Figure 7). Similarly, transcription levels of these genes were highest in the proximal segment of DAG4 cotyledons (Figure 6B), while the levels of *ARR1* and *WIND1* genes were not significantly different between the segments (Figure 8). Although initial transcript levels of *CYCD3-1*, *STM*, and *WUS* were lower in cotyledon explants of DAG4 than in DAG7 or DAG10 seedlings, incubation in SIM resulted in increased expression of these genes. We could not identify the molecular mechanism underlying the increase in *CYCD3-1*, *STM*, and *WUS* in DAG4 cotyledon explants with the current data. However, we could confirm that temporal increases in transcript levels of these shoot regeneration regulatory genes in the hypocotyl and the cotyledon explants led to the acceleration of adventitious shoot formation.

Expression of *WIND1-4* genes is strongly up-regulated within a few hours of wounding, and the ectopic overexpression of individual *WIND* genes is sufficient to induce callus formation in *Arabidopsis* [26]. Four type-B ARR transcription factors, *ARR1*, *ARR2*, *ARR1*0, and *ARR12*, play essential roles in shoot regeneration in *Arabidopsis*. However, we found that the transcript levels of *ARR1* and *WIND1* in DAG4 hypocotyl and cotyledon explants of tomato did not significantly increase over time (Figure 7 and Figure 8). It is not clear why the transcript levels of *ARR1* and *WIND1* did not respond significantly during shoot regeneration. Iwase et al. (2011) showed that the transcription level of *WIND1* was rapidly up-regulated within a couple of hours after wounding in *Arabidopsis* seedlings but did not change after the application of exogenous auxin or cytokinin [26]. Furthermore, Jones et al. (2011) showed that there was no early response in *ARR1* transcripts levels in root tips of DAG7 *Arabidopsis* (Col-0) incubated with 5 μM of trans-Zeatin in a liquid medium, but they were down-regulated at later time points [36]. Besides, the expressions of a wide variety of cell-cycle genes in cultured suspension cells of *Arabidopsis* were differently induced by auxin, cytokinin, and sucrose combinations. Transcripts of cyclin-dependent kinase (CDK)-encoding genes *CDKA;1* and cyclins of A- (*CYCA2;1*) and D-types (*CYCD2;1* and *CYCD4;1*) were induced by sucrose in *Arabidopsis* cell suspension [37,38], whereas *CDKB1;1* and *CYCB1;1* were induced most strongly by the combined administration of auxin and cytokinin [38]. Soni et al. (1995) noted that *CYCD3;1* was most strongly induced by cytokinin alone [39], but Rhichard et al. (2002) reported the same with cytokinin, auxin, and sucrose together [38]. Moreover, *WUS* induction was regulated by the ratios of cytokinin/auxin during the inflorescence differentiation [40]. *WUS* signals were observed at 20 mg L^−1^ Zeatin with 0.01 mg L^−1^ IAA and 2 mg L^−1^ Zeatin with 0–1 mg L^−1^ IAA, but *WUS* expression was not induced under 0, 0.02, or 0.2 mg L^−1^ Zeatin with 0.01 mg L^−1^ IAA [40]. This suggests that *ARR1* and *WIND1* may have different gene expressions depending on shoot regulators such as auxin or cytokinin or on the carbon source included in the SIM.

According to the results obtained, the temporal and the positional differences in gene-expression profiles from hypocotyl and cotyledon explants of tomato suggest that increased expression levels of the major shoot-regulating are associated with adventitious shoot formation. However, additional genetic and molecular properties are required to understand the correlation between these potentially major regulatory factors and plant-regeneration capacity. In addition, further studies are needed to see whether other tomato genotypes produce the same results.

In summary, we established an efficient and reproducible shoot-regeneration system for tomato explants through the optimization of culture conditions and explant preparation. Furthermore, we also propose that temporal and spatial differences in the expression of *CYCD3-1*, *STM*, and *WUS* can affect shoot organogenesis in tomato explants. The plant-regeneration system established in this study could be applied to diverse fields of research, including genetic transformation and gene editing for tomato quality improvement.

## 4. Materials and Methods

### 4.1. Plant Materials and Seeds Germination

Tomato (*Solanum lycopersicum* cv. Micro-Tom) seeds were separated from the harvested tomato, soaked in 1.2% protease solution (Rapidase) for 1.5 h, and then rinsed with water. Subsequently, the seeds were soaked in 1.6% sodium hypochlorite (NaOCl) for 10 min, rinsed in water, dried thoroughly, and stored until use.

The seeds were sterilized in 70% (*v*/*v*) EtOH for 1 min, soaked in 0.8% NaOCl solution for 1–3 min, and then rinsed thoroughly with sterilized, distilled water. After surfacesterilization, the seeds were kept in the dark for an initial 3-day period at 4 °C and thereafter placed onto Murashige and Skoog (MS) basal salt mixture (Duchefa, The Netherlands) supplemented with 3% (*w*/*v*) sucrose, 0.4 mg L^−1^ thiamine, 100 mg L^−1^ myo-inositol, and 0.4% (*w*/*v*) Gelrite, pH 5.8 with 1 N KOH, and cultured under 70% relative humidity and light at approximately 30 µmol m^−2^ s^−1^ (light/dark regime of 16/8 h) at 25 °C.

### 4.2. Culture Conditions for Adventitious Shoot Formation from Tomato Explants

To determine optimal shoot regeneration and gene expression in cotyledon and hypocotyl explants, 4-, 7-, and 10-day-old seedlings were excised after germination (hereafter referred to as day-after-germination: DAG4, DAG7, and DAG10, respectively), divided into three segments, and cultured onto shoot induction medium (SIM) (MS basal salt mixture (Duchefa, The Netherlands)) containing several combinational treatments of 1–2 mg L^−1^ Zeatin, 0.1–0.2 mg L^−1^ IAA, 2% or 3% sucrose, 0.4 mg L^−1^ thiamine, 100 mg L^−1^ myo-inositol, and 0.4% (*w*/*v*) Gelrite, pH 5.8 with 1 N KOH for the indicated time at equal intervals (approximately 2 cm distance). For each treatment, three replicates of nine cotyledon explants were placed in a Petri dish (90 × 90 mm) except for those used for a density experiment (Figure S3). All cotyledons were tested in all abaxial orientations except for those used in an orientation experiment (Figure S2). All cultures were incubated under 70% relative humidity and light at approximately 30 µmol m^−2^ s^−1^ (light/dark regime of 16/8 h) at 25 °C. The efficiency of adventitious shoots formation was calculated with the ratios from the total number of explants and the number of explants showing shoot formation.

### 4.3. RNA Isolation and Quantitative Reverse Transcription PCR (qRT-PCR) Analysis

Total RNA was prepared from hypocotyl and cotyledon explants that were grown in the same culture conditions. Hypocotyl and cotyledon explants from 4-, 7-, 10-day-old seedlings were collected. These explants were then cut into three segments and incubated in a shoot induction medium (MS basal salt mixture (Duchefa, The Netherlands)) supplemented with 1 mg L^−1^ Zeatin, 0.1 mg L^−1^ IAA, 3% (*w*/*v*) sucrose, 0.4 mg L^−1^ thiamine-HCl, 100 mg L^−1^ myo-inositol, and 0.4% (*w*/*v*) Gelrite, pH 5.8 with 1 N KOH for 4, 8, and 12 days. Total RNA was prepared using an Aqueous Kit (Ambion) with a TURBO DNA-Free Kit (Applied Biosystems). cDNA was synthesized using a high-capacity cDNA Reverse Transcription Kit (Applied Biosystems).

To examine the transcript levels of *ARR1*, *CYCD3-1*, *STM*, *WIND1*, and *WUS* in the individual explants, each primer was designed using the Real-time PCR tool OligoAnalyzer 3.1 (Intergrated DNA technologies, Inc. USA). The qRT-PCR was performed using an SYBR Green Kit (BioFACT, Korea) to detect the transcript levels of each gene on the CFX96 ^TM^ Real-Time PCR Detection System (Bio-Rad). The primers used for PCR were as follows: 5′-AAGCCCCTCTTAACCCAAAG-3′ and 5′-ACCAGAGTCCAACACAATACC-3′ for *Actin4*; 5′-GCAAATGGATTCTGGGACAATG-3′and 5′-CCCTCCCAATCCGTTTCATC-3′ for *ARR1*; 5′-CAAGGAGAAGGTGGAAGGATG-3′ and 5′-CTGTTGGACTCCCTGGTAATG-3′ for *CYCD3-1*; 5′-GCGAGGCAATGGATAGAAATG-3′ and 5′-TTAAGGCTTCCCAAGTAACCG-3′ for *STM*; 5′-CAAGCCCGTTTCCATGAAG-3′ and 5′-AATGTACCTAGCCAAAGCCG-3′ for *WIND1*; 5′-TGGAACTTTGGCTATGGAGAAG-3′ and 5′-GGGTAAGTTGCTGGAGAAGTAG-3′ for *WUS*. *Actin4* (*ACT*4) was used as an internal control.

### 4.4. Statistical Analysis

Experiments were formed at least three times. Data were analyzed using the one-way analysis of variance (ANOVA) test, followed by Tukey’s multiple comparison test. Differences were considered statistically significant when *p* < 0.05.

## Data Availability

All datasets for this study are included in the manuscript and the supplementary files.

## Supplementary Materials

Supplementary materials can be found at https://www.mdpi.com/1422-0067/21/15/5309/s1. Figure S1. Tomato seedlings at 4-, 7-, and 10-days-after germination grown in Murashige and Skoog (MS) medium. Figure S2. Effect of orientation of cotyledon explants in the shoot induction medium (SIM) for adventitious shoot formation. Figure S3. Effect of the different number of cotyledon explants in shoot induction medium (SIM) for adventitious shoot formation.

## Accession Numbers

Sequence data from this article can be found in the Pyotozome v2.1 under the following accession numbers: *ARR1* (Solyc07g009560.1), *CYCD3-1* (Solyc04g078470.2), *STM* (solyc02g081120.2), *WIND1* (Solyc12g056980.1), *WUSCHEL* (Solyc02g083950.2.1), and *Actin 4* (Solyc04g011500.2).
