# Supplementary material for: Temporal and Spatial Expression Analysis of Shoot-Regeneration Regulatory Genes during the Adventitious Shoot Formation in Hypocotyl and Cotyledon Explants of Tomato (CV. Micro-Tom)
Source: Int J Mol Sci. 2020 Jul 26;21(15):5309. doi: 10.3390/ijms21155309 (PMC7432687; doi:10.3390/ijms21155309)
Supplement: Supplementary file 1 [file ijms-21-05309-s001.pdf]

Supplementary data

Temporal and spatial expression analysis of shoot-regeneration regulatory genes during the adventitious shoot formation in hypocotyl and cotyledon explants of tomato (cv. Micro-Tom)

Myoung Hui Lee[1], Jiyoung Lee[1], Eun Yee Jie[1], Seung Hee Choi[1], Lingmin Jiang[1,2], Woo Seok Ahn[1,3], Cha Young Kim[1], and Suk Weon Kim[1*]

[1]Biological Resource Center, Korea Research Institute of Bioscience and Biotechnology (KRIBB), Jeongeup 56212, Republic of Korea.
[2]Department of Bioactive Materials, Chonbuk National University, Jeonju 54896, Republic of Korea.
[3]Department of Bioenergy Science and Technology, Chonnam National University, Gwangju 61186, Republic of Korea.

*** Correspondence:**
Suk Weon Kim
kimsw@kribb.re.kr

Tel: +82-63-570-5650; Fax: +82-63-570-5609

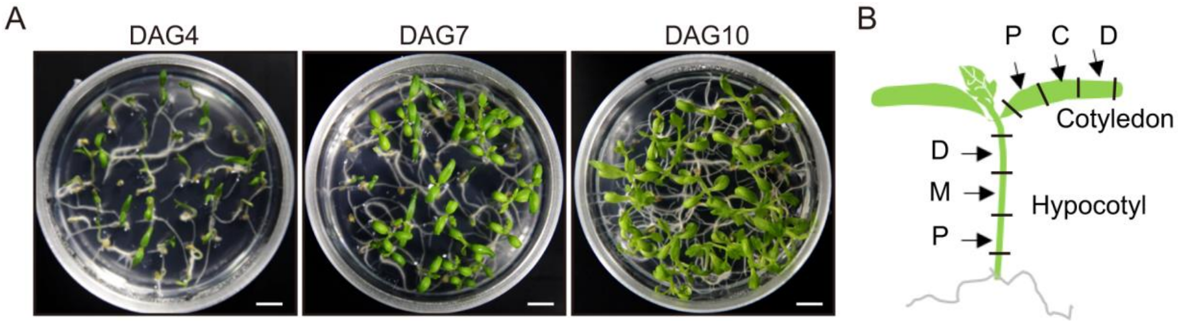

Figure. S1. Tomato seedlings at 4-, 7-, and 10-days-after germination grown in Murashige and Skoog (MS) medium.

(**A**) DAG4, DAG7, and DAG10 seedlings in MS medium. Scale bar = 1 cm. (**B**) The Distal (D), middle (M), and Proximal (P) position of hypocotyl explants and the proximal (P), central (C), and Distal (D) position of cotyledon explants. DAG = day after germination. Scale bar = 1 cm.

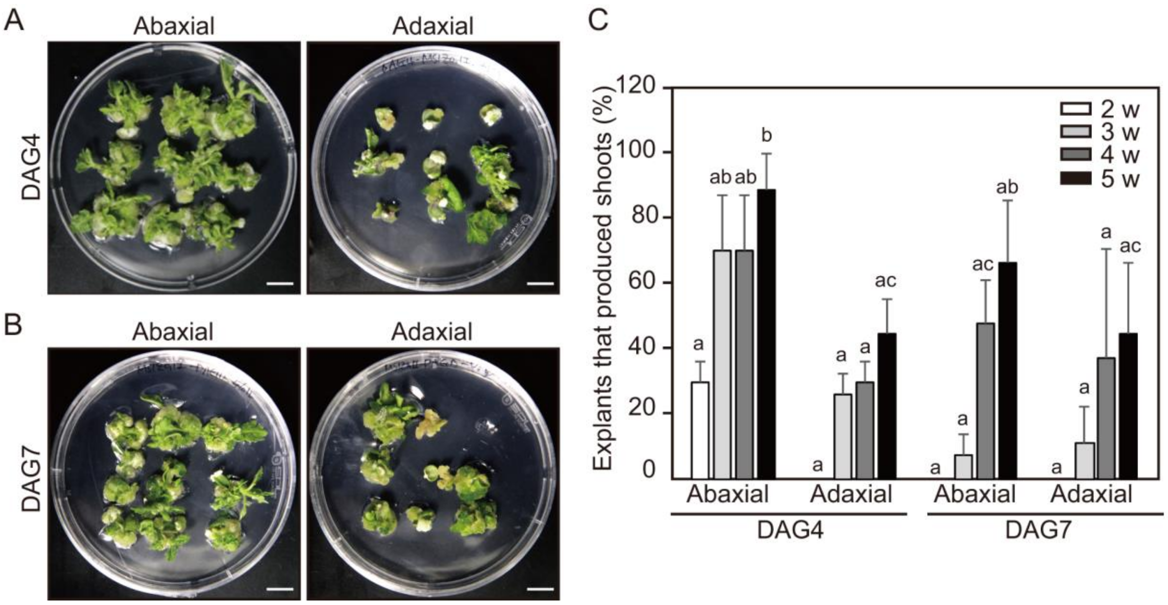

Figure. S2. Effect of orientation of cotyledon explants in the shoot induction medium (SIM) for the adventitious shoot formation.

(**A**, **B**) Different orientation of cotyledon explants of DAG4 (**A**) and DAG7 (**B**) seedlings incubated for four weeks. Scale bar = 1 cm.

(**C**) The efficiency of adventitious shoot formation at different orientation of cotyledon explants. Three independent experiments were performed on 81 explants. Different letters on bars indicate significant differences between each treatment (ANOVA followed by a Tukey's test, $P < 0.05$). Error bars represent SD (N = 81). DAG = day after germination; w = weeks.

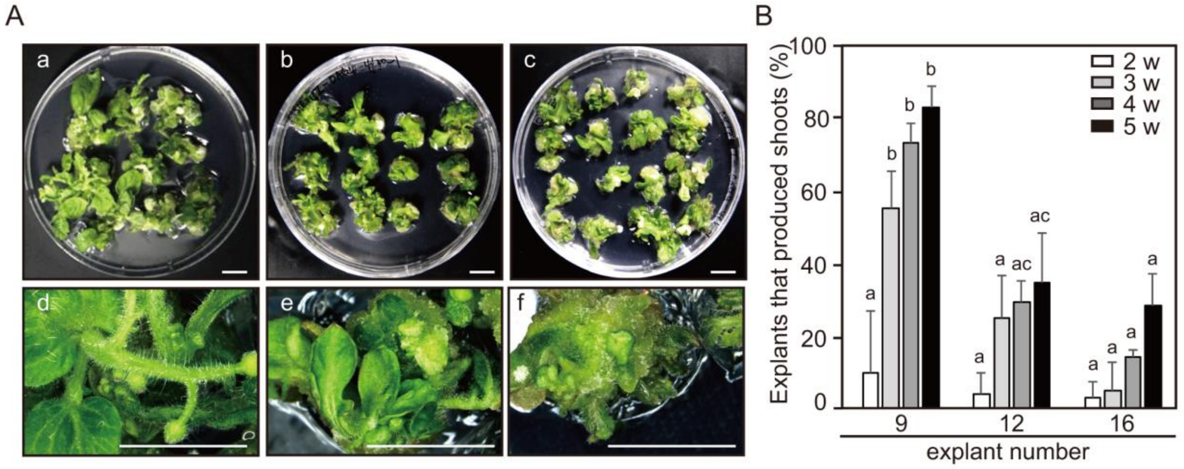

Figure. S3. Effect of the different number of cotyledon explants in shoot induction medium (SIM) for the adventitious shoot formation.

(**A**) Different numbers of cotyledon explants of DAG4 seedling in SIM. Cotyledon explants show four weeks (a-c) and five weeks (d-f) after incubation. The cotyledon explants number were 9 (a and d), 12 (b and e), and 16 (c and f) on a plate. Scale bar = 1 cm.

(**B**) The efficiency of adventitious shoot formation at a different number of cotyledon explants. Three independent experiments were performed on 81 explants. Error bars represent SD (N = 81). Different letters on bars indicate significant differences between each treatment (ANOVA followed by a Tukey's test, $P < 0.05$). DAG = day after germination; w = weeks.
